# Lichen Planus of the Lip—Case Series and Review of the Literature

**DOI:** 10.3390/medicina60060987

**Published:** 2024-06-16

**Authors:** Corina Andreea Marcu (Selaru), Ioanina Parlatescu, Serban Tovaru, Carmen Larisa Nicolae, Mariana Costache, Mihaela Tovaru

**Affiliations:** 1 Doctoral School, “Carol Davila” University of Medicine and Pharmacy, 020021 Bucharest, Romania; corina.selaru@drd.umfcd.ro; 2 Faculty of Dentistry, “Carol Davila” University of Medicine and Pharmacy, 020021 Bucharest, Romania; serban.tovaru@gmail.com (S.T.); carmen-larisa.nicolae@umfcd.ro (C.L.N.); mihaela.tovaru@umfcd.ro (M.T.); 3 Faculty of Medicine, “Carol Davila” University of Medicine and Pharmacy, 020021 Bucharest, Romania; mariana.costache@umfcd.ro

**Keywords:** lip lichen planus, oral lichen planus, lip lesions

## Abstract

*Background and Objectives*: Lichen planus of the lip (LPL) is a chronic inflammatory condition that resembles actinic cheilitis, discoid lupus erythematosus, graft-versus-host disease, and lichenoid reaction to dental materials or drugs. The purpose of this study was to conduct a literature review on lichen planus lip involvement and to report a retrospective observational study that characterises and explores the clinical, histopathological, and evolution of the lesions in a group of patients with unique involvement of LPL. *Materials and Methods*: Clinical data of patients diagnosed with LPL was retrieved from the medical charts of the patients referred to the Oral Pathology Department of the “Carol Davila” University of Medicine and Pharmacy. A concurrent electronic literature research was carried out using PubMed and Web of Science from 2003 to 2023. *Results*: Eleven patients diagnosed with unique LPL were analysed (male/female ratio was 1.75, mean age 63.64 years ± 12.52). All patients presented lesions of the lower lip; the clinical forms were atrophic (six cases) and erosive (five cases), and the histopathological exam confirmed the diagnosis. After topical treatment with corticosteroids, most of the patients had complete remission. The literature review revealed 24 studies (sixteen case reports and eight case series) which comprised 84 patients. Isolated lip involvement was reported in 17 studies, and five articles with concomitant oral lichen planus, while two articles did not mention this criterion. *Conclusions*: Our study brings new data on isolated lichen planus of the lip that primarily affects the lower lip with predominance in male patients. It was reported worldwide in patients between 22 and 75 years old. Topical corticosteroids were the main treatment prescribed and they usually brought remission of the lesions. Lichen planus of the lip is a challenging diagnosis for oral health practitioner providers as well as for dermatologists.

## 1. Introduction

Lichen planus (LP) is a chronic inflammatory mucocutaneous condition with an unknown etiopathogenesis [1]. LP primarily affects the skin and oral mucosa. However, other mucous membranes can be affected (genital, oesophageal, and conjunctival mucosa), but different areas of the skin (such as the scalp and nails) can also be affected [2]. Cutaneous LP lesions are usually self-limiting and may sometimes cause itching. Oral lesions of LP are chronic and rarely go into remission. The potentially malignant nature of oral lesions often forms the basis for morbidity [2]. Oral lichen planus (OLP) affects about 0.1% to about 4% of the middle-aged population and has a definite female gender predilection [3]. It usually presents bilaterally on the buccal mucosa, dorsum of the tongue, and the gingival mucosa [3].

The predisposition of lip lesions to various traumas (biting the lips, applying make-up, or exposure to sunlight) changes their clinical appearance, thus mimicking lesions of a diverse nature and putting weight on their correct diagnosis. Lichen planus of the lip carry a malignant potential, thus requiring prompt diagnosis and management of these lesions [1]. Moreover, there are cases where the lips are the only site affected by lichen planus, entitled lichen planus of the lip (LPL), and a wide range of lip diseases must be considered for differential diagnosis.

The chronic lip manifestations may resemble actinic cheilitis, discoid lupus erythematosus, graft-versus-host disease, or lichenoid reaction to dental materials or drugs, which makes us consider these conditions for the differential diagnosis [4]. Recurrent herpes, exfoliative cheilitis, and erythema multiforme should be taken into consideration if the lesions are recent [4].

Actinic cheilitis (AC) is a potentially malignant disorder of the lower lip due to chronic exposure to ultraviolet radiations. Clinically, there are areas of erythema, keratosis, scaling, and dryness, and it is difficult to distinguish between the limit of the lips and the skin. Plaques, ulcerations, and crusts may appear in more advanced cases. The lesion may progress to squamous cell carcinoma if treatment is not set [5]. Individuals, mainly men with light skin who work outside (farmers, fishermen, builders, and sportsmen) are most affected by this condition [6]. Additionally, it is more frequently noticed in men as they utilise lip balm less frequently than women do [7]. Because the lesion progresses slowly and normally shows no symptoms, it is sometimes mistakenly associated with ageing conditions [5]. The primary risk factor for AC is chronic sunlight exposure. Other factors linked to AC development are the patient’s age, genetic susceptibility, geographic latitude of residency, outdoor occupation, leisure activities, and lack of usage of lip protectants [7].

The second disease to be taken into account for differential diagnosis of LPL is discoid lupus erythematosus (DLE). DLE is a chronic mucocutaneous disease that usually affects sun-exposed areas and presents with persistent erythema and discoid plaques. About 20% of cases affect the oral mucosa, with the lower lip being among the most frequently affected areas [8]. Biopsies from DLE lesions are the best option because normal tissue biopsies, particularly from untreated areas, frequently yield negative results. A biopsy of normal tissue that yields a positive result suggests that DLE may progress to systemic lupus erythematosus (SLE) [9]. The direct immunofluorescence evaluation detects the “lupus band” present at the basement membrane level for lesions of more than 6 weeks duration and lesions of the head, neck, and upper extremities rather than the trunk [10]. The severity of DLE might be determined by antinuclear antibody (ANA) titer assessed via indirect immunofluorescence. A high ANA titer serves as a reminder to adjust treatment regimens to prevent the development of systemic lupus erythematosus and is one of the risk factors for the development of DLE in SLE patients [9].

Another condition that can present lesions mimicking LPL is graft-versus-host disease (GvHD) which is a complication of bone marrow transplants or allogeneic hematopoietic stem cell therapy. This is a multi-organ disease that manifests as a range of signs and symptoms and the oral mucosa is one of the most common areas involved. The primary complaint is discomfort during eating. White stria and/or plaques and erosive and ulcerative oral cavity areas are the disease’s initial signs [11]. The buccal mucosa and lateral edges of the tongue are usually affected, and the dorsal surface of the tongue may show papillary atrophy. Other clinical features include xerostomia (dry mouth), and patients may develop recurrent mucoceles. Malignant transformation has been reported in subsequent studies [7].

Oral lichenoid lesions (OLLs) are intraoral lesions that have a reticular and/or striated keratotic, atrophic appearance or even ulcers. Their clinical symptoms are comparable to OLP, but OLLs are caused by a different agent. The term “oral lichenoid reactions” (OLRs) is used synonymously. These conditions are divided into three categories: (1) topographically related to a dental restoration, usually amalgam; (2) drug-related; and (3) associated with GvHD [12]. The location of OLLs/OLRs related to hypersensitivity to dental restorations is typically the nearby area with the allergenic material, whereas OLP usually presents as a bilateral condition, particularly on the buccal mucosa. Drug-induced OLLs/OLRs have a variety of clinical characteristics, with a tendency to be unilateral and erosive. When necessary, a skin patch test [13], a careful history of the disease, clinical examination, and histopathological examination are used to establish the diagnosis of OLP, OLLs, and OLRs. Even though reactions may take weeks or months to manifest, the history of any association between the beginning of drug intake and the onset of symptoms may also be instructive in cases of OLLs/OLRs suspected of being drug-induced. Nonetheless, it can be challenging to discern between OLLs/OLRs and OLPs under the microscope, and pathologists are divided on how to do so. The first reports of a potentially malignant change in OLLs were made in a series of Dutch cases [14].

As a number of disorders mimic clinical LPL and raise difficulties in diagnosing this condition, we conducted a retrospective study and a review of the literature on this topic. The objective of the present observational study was to describe and analyse the clinical, histopathological presentation, and evolution of unique lip involvement of a case series of lichen planus patients.

## 2. Materials and Methods

### 2.1. Study Design

We performed an observational retrospective study of 11 patients diagnosed with lichen planus of the lip as a unique involvement. The study was carried out in the Oral Pathology Department of the Faculty of Dentistry, “Carol Davila” University of Medicine and Pharmacy, Bucharest. The patients were admitted from 2004 to 2023.

The selection criteria for this study included the presence of the following data in the medical chart: detailed clinical description of the lesions (type, location and size), histopathological confirmation of the OLP, results of blood tests, and informed consent signed. We excluded patients with concomitant lesions on the oral mucosa and lip.

For the present study, we also collected demographic data on patients (age, gender, background), smoking status, other associated diseases, and presence of skin lesions. The anamnestic data regarding the symptoms (asymptomatic, pain, or burning) and the onset of the lesions were also recorded. Depending on the patient’s report, stress was categorized as either present or absent. The clinical forms of OLP were classified according to AAOM recommendations [15]. The 6th classical clinical patterns of OLP comprise reticular, papular, plaque lesions, atrophy, erosions/ulcers, and bullous type [3]. In this study we categorised the clinical forms of OLP in 4 patterns: keratotic type which included white reticular, papular or plaques lesions, atrophic type which included erythematous lesions surrounded by white components, erosive/ulcerative, and bullous form [15].

To provide a consistent indication of OLP severity, we scored the lesions using the Thongprasom score, commonly used by experienced oral pathologists [16]. This score has a scale from 0 to 5: score 0—no lesions, score 1—mild white striae, score 2—white striae and atrophic area less than 1 cm^2^, score 3—white striae and atrophic area more than 1 cm^2^, score 4—white striae and erosive area less than 1 cm^2^, score 5—white striae with erosive area more than 1 cm^2^ [17,18].

For an accurate diagnosis, we used the histopathological evaluation of OLP lesions in all patients, as recommended by the American Academy of Oral and Maxillofacial Pathology [15]: in the epithelium hyperpara/hyperorthokeratosis, Civatte bodies, basal cell hydropic alteration, and in the underlying chorion a band-like predominant lymphocytic infiltrate. In patients with antihypertensive drugs (beta-blockers and angiotensin-converting enzyme inhibitors), the lichenoid drug reaction was excluded by the histopathological evaluation [3]. When compared to OLP, the lichenoid lesions exhibit a more diffuse infiltrate, with eosinophils, plasma cells, and a higher number of colloid bodies [3].

The outcome measures were divided into partial or complete remission and aggravation. The initial and final Thongprasom score was calculated.

This study was performed according to the recommendations of the Declaration of Helsinki and was approved by the University Ethics in Research Committee (No. 1140/13 January 2023). Written informed consent was obtained from all the patients.

### 2.2. Review Design

Electronic searches of articles written in English were performed using the databases of PubMed and Web of Science with publications from 2003 to 2023. Reference lists were also manually searched to identify additional studies. The keywords we used are: oral lichen planus, lichen planus lip.

The inclusion criteria were the relevance of the title or the abstract for the research topic. The exclusion criteria were: (1) incomplete or missing clinical description of the lesions, (2) other lip diseases mimicking lichen planus of the lip, and (3) narrative articles.

The following data have been taken into account for each article that reports a case or a case series of LPL: the year of publication, number and nationality of patients, age and gender, single lip involvement or associated OLP lesions, main symptoms, clinical aspects, presence of cutaneous lesions, associated general diseases, histopathological evaluation, therapy, and evolution.

After selecting the papers, three authors (C.A.M, I.P., C.L.N.) extracted and categorised the data. A fourth author (T.S.) verified the selection and classification of the articles.

## 3. Results

The study cohort consists of 11 patients diagnosed with unique lip involvement of LP. We found a male predominance in our study (seven men and four women). The mean age was 63.64 years, 12.52 ± SD (women’s mean age of 54 years and men’s mean age of 69.14 years). Four patients were former smokers. No patient reported LP lesions in their family members.

Regarding the associated comorbidities, three patients had hypertension, one hepatitis C, and three of them did not suffer from any other condition.

Most of the patients (six out of eleven) presented in our department for aesthetic complaints, followed by three patients with burning sensations and two with pain.

No patient presented skin or other mucosal sites of lichen planus lesions.

**Figure 1:**
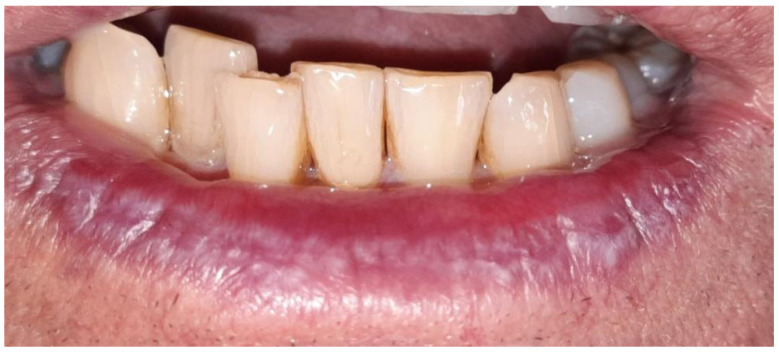
Atrophy and white striae of the lip lichen planus.

**Figure 2:**
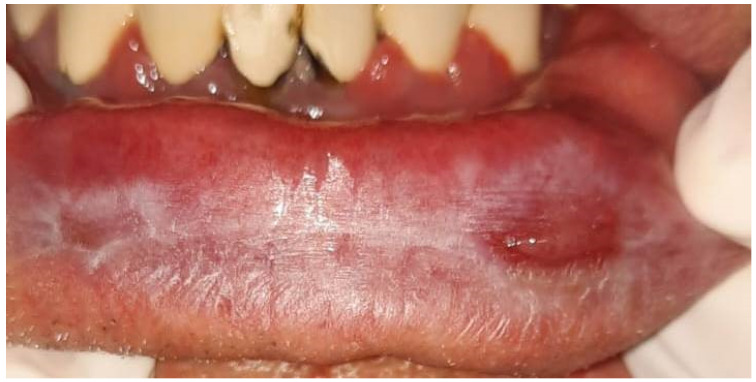
Erosive lesions of the lower lip lichen planus.

In all the patients the lichen planus lesions involved the lower lip. The clinical forms encountered were atrophic (Figure 1) in six patients and erosive in five patients (Figure 2).

The initial clinical Thongprasom score ranged between 2 to 5, with a mean value of 3.18 and a standard deviation of ±1.07.

**Figure 3:**
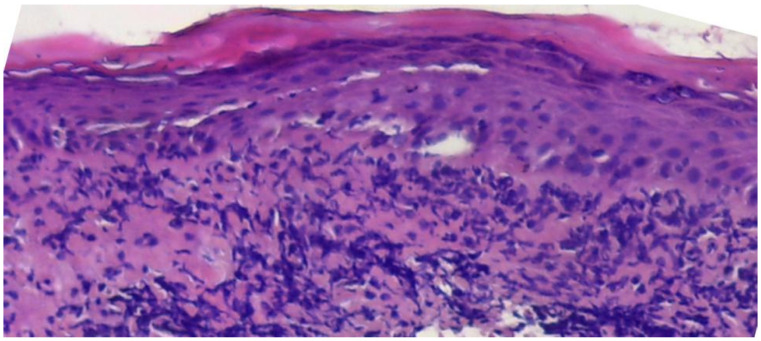
Histopathological appearance of the lower lip LP. The epithelium presents an area of orthokeratosis; in some places the cells of the basal layer are missing, and an inflammatory lymphocytic infiltrate organised in the subepithelial band is observed in the superficial chorion. Hematoxylin–eosin staining, 100X.

In all cases, the diagnosis was confirmed by the biopsy (Figure 3).

Treatment for five patients included clobetasol propionate 0.05%, methylprednisolone aceponate 0.01% with hydrating lip balm in three cases, fluocortolone 0.25% in cream in two patients, and fluocinolone acetonide 0.01% in orabase in one case. Ten patients attended follow-ups for an average of eight months, and one patient failed to return for control.

After the treatment, most of the lesions showed signs of complete remission in eight patients, in two cases were presented aggravated lesions, and one patient with no reported outcome (no follow-up).

Demographic characteristics of the study population are included in Table 1.

In the present literature review, we included 24 descriptive studies from 13 different countries across Asia, Europe, Africa, and North America that met our inclusion criteria. These studies comprised 84 patients (51 males and 33 females).

Seventeen reports included studies only with LPL as isolated involvement of the lips, five articles with concomitant OLP, and two articles did not mention this criterion.

The age of the patients ranged from 22 to 75 years and the female/male ratio was 0.64. For the case reports [2,19,20,21,22,23,24,25,26,27,28,29,30,31,32,33], the mean age was 50.81 years, SD ± 11.26, and for the cases series [1,34,35,36,37,38,39,40] it was 53.50 years, SD ± 16.06. As for the geographical distribution, seventeen reports were from Asia, five from Europe, one from Africa, and one from the USA.

In the analysed studies the most frequent symptoms were pain and bleeding in six studies out of twenty-four, pain in five studies, and burning symptoms were reported in four studies.

The most frequently reported clinical form of LPL was the erosive one in fourteen studies and plaque lesions in eight studies. Confirmatory biopsies were mentioned in most of the studies except two reports [1,34].

The histopathological examination reveals the most common diagnosis as lichen planus of the lip, except one study which reports Nivolumab-related LPL.

In the analysed studies, the treatment of the lesions consisted of the application of topical corticosteroids [1,2,22,23,24,25,28,32,33,34,37,39], immunosuppressive creams [30,31,36,40], and topical corticosteroids associated with immunosuppressive creams [19,20,27,38], except one study [26] where the patient received traditional Chinese medicine.

Regarding the treatment result, in twelve studies [22,23,24,25,26,27,30,32,33,36,39,40] all of the patients showed remission, in three studies [1,2,20] regression and recurrence, in one study [19] improvement after replacing secukinumab with risankizumab, and in one [28] no improvement followed by malignization after 6 years. Lehner et al. [34] reported improvement in twelve patients and no improvement in two patients in their study with twenty-three patients. Garma et al. [37] reported two cases where the male showed remission and the female showed regression and recurrence of the lesions. In the study by Gencoland et al. [38], three of the four patients included in the study showed remission, and one case presented regression and recurrence.

## 4. Discussion

Since LP involvement of the lip alone was first mentioned in 1961 by Altman and Perry [41], there have been many cases reported in the literature [34,38,39].

The current retrospective study describes the clinical, pathological, and evolutive patterns of 11 patients with unique lip involvement of LP from Bucharest, Romania. Few studies report only the unique lip involvement of LP, and these are case reports [2,10,21,22,23,28,29,30,31,32,33] or case series [35,38,39]. In this case series report, we analysed and compared our findings primarily with those of other European studies. All our patients presented lesions located only on the lower lip, although other authors also reported both lips [21,39] but with a preponderance of the lower lip [1].

Compared to oral lichen planus, which is more prevalent in female patients [15], we observed a predominance in male patients of the isolated lesions of lip lichen. Other authors [1,39] found similar findings; however, Lehner et al. [34] observed lichen of the lip more frequently in women.

In our series, the mean age was 63.64 years, in concordance with other reported studies from France [34] and Italy [1,39].

Regarding the association of systemic diseases in our study group, we found an association with hypertension (three patients), vitiligo (two patients), hypothyroidism (one patient), and hepatitis C (one patient), while Nuzzolo et al. [1] reported the presence of hepatitis C infection in isolated lip lesions and concurrent OLP lesions.

Cutaneous LP is frequently linked to hepatitis B, hepatitis C, hepatitis B vaccine, primary biliary cirrhosis, and other autoimmune diseases such myasthenia gravis, alopecia areata, vitiligo, morphea, or ulcerative colitis [42,43]. Many concurrent medical conditions, including hypertension, diabetes, metabolic syndrome, thyroid disorders, psychological conditions, chronic liver disease, gastrointestinal disorders, and genetic predisposition to cancer, are associated with oral lichen planus [44]. As previously reported, dyslipidemia—a risk factor for cardiovascular diseases—is associated with both cutaneous lichen planus [44] and oral lichen planus [45]. Therefore, the recommendation to prevent cardiovascular diseases by monitoring through investigations and reducing risk factors is important for patients diagnosed with LP.

The lips are an area at the border between the skin and oral mucosa and are covered in a specialised epithelium. Lichen planus of the lip represents a challenging diagnostic for dentists, general practitioners, oral pathologists, and dermatologists, especially when these lesions are not accompanied by specific oral mucosal lesions. The aetiology, pathogenesis, and evolution of oral lichen planus and cutaneous lichen planus have differences [15,42].

The clinical pattern of LLP includes keratotic lesions with stria distribution, atrophy, erosions, and ulcers. Six patients in our study group had atrophic forms, and five had erosive forms; in contrast, the majority of literature reviews revealed erosive and keratotic plaque-like lesions (see Table 2).

This current research’s addition are the data evaluating the intensity of LPL lesions performed with the Thongprasom score as OLP and LPL show the same lesional pattern. Initially, this score was thought to evaluate the effectiveness of the OLP treatment [17]. The mean of our initial score was 3.18 with SD 1.07, while a study from 2021 reported erosive OLP in 40 patients with a mean score of 3.78 (1.30) [46].

Dermatoscopy was described as a method of investigation the lip vermillion for distinguishing between actinic cheilitis, discoid lupus erythematosus, graft-versus-host disease (GvHD), and lip lichen [21,25,47]. It adds characteristics to the colour, distribution of lip structures, shape, and distribution of the vessels that are not apparent during a clinical examination [46]. Wickham striae can be identified in lichen planus, while telangiectasia, dark pigment patches, and white, structureless areas are detected in lupus. Anastasia et al. [46] examined the dermatoscopic characteristics of sixteen cases of vermilion chronic GvHD and determined that, despite three cases showing similar appearances, this examination helps differentiate it from LLP.

A case of lower lip cancer-like mimicking LLP with unusual features (verrucous, hyperkeratotic plaque) is reported by Mozafari et al. [20]. The patient received oral triamcinolone, topical tacrolimus 0.1%, and clobetasol ointment as local treatment. Oral prednisolone and mycophenolate mofetil were used as general treatments after the remission of the lesions [20].

The clinical aspect between OLP and OLL is clinically similar and difficult to differentiate even histologically. In a four-year study of 23 patients with lip lesions of OLP and OLL, Lehner et al. [34] found no correlated dental factor.

As for OLP diagnosis, the fulfilment of both clinical and histopathological criteria was proposed [15], and the presence of both criteria is useful for LPL. The clinical criteria for OLP diagnosis are the presence of reticular keratosis with symmetric distribution on buccal mucosa and histopathological criteria which include a predominant lymphocytic band-like infiltrate, with liquefaction degeneration of basal cells and no dysplasia [48]. The clinical pattern of LPL associates reticular or plaque-like lesions as well as atrophy, erosions, and ulcers. We emphasize that the lip’s epithelium is thinner than the oral epithelium, which might cause issues in diagnosing and interpreting histopathology. Other diagnostic methods such as dermatoscopy or in vivo reflectance confocal microscopy may bring Supplementary Data for this condition. In vivo reflectance confocal microscopy is used for AC and its progression to carcinoma [49].

One limitation of the current research is the lack of a control group in this case series study; nonetheless, this approach offers comprehensive descriptive data for an uncommon lichen planus presentation. Although the study’s design does not aim to evaluate the effectiveness of the treatments, every case’s treatment strategy and outcome were described. This topic requires further investigations, particularly regarding the treatment outcome in isolated LPL versus LPL concomitant with OLP.

## 5. Conclusions

Our study brings new data on isolated lichen planus of the lip that primarily affects the lower lip with predominance in male patients. It was reported worldwide in patients between 22 and 75 years old. The erosive and atrophic forms of LPL caused burning sensations, pain, and aesthetic complaints. Topical corticosteroids were the main treatment prescribed and they usually brought remission of the lesions. Lichen planus of the lip is a challenging diagnosis for oral health practitioner providers as well as for dermatologists.

## Figures and Tables

**Table 1:** Demographic characteristics and clinical data of the patients included in the present study.

Case no.	Year of Presentation	Sex	Age	Environment	Symptoms	Stress	Medical History	Drugs	Self Reported Onset (Months)	Clinical Form	Score Thongprasom Evolution (Initial to Final)	Treatment	Outcome and Follow-Up Period
1	2012	F	64	U	Pain	No	Hepatitis C	No	3	Atrophic	2 to 0	Clobetasol propionate 0.05%	Remission in 5 months
2	2013	M	65	R	Burning	No	Hypertension	Antihypertensives	12	Atrophic	2 to 0	Clobetasol propionate 0.05%	Remission in 6 months
3	2012	M	83	U	Aesthetic complaints	Yes	Hypertension, Arteriosclerosis, Maxillary sinusitis	Statins, Antihypertensives	6	Erosive	4 to 5	Clobetasol propionate 0.05% and Nystatin in orabase	Aggravated, OLP lesions in 2 years
4	1994	M	67	U	Burning	No	Hypertension, Nodular tuberculosis, Rheumatoid arthritis	No	72	Atrophic	3 to 0	Fluocinolone acetonide 0.1% in orabase	Remission in 6 months
5	2004	F	66	U	Aesthetic complaints	No	Vitiligo	No	4	Atrophic	2 to 0	Clobetasol propionate 0.05%, Fluocortolone 0.25% in cream	Remission in 7 months
6	1984	M	60	R	Aesthetic complaints	No	No	No	3	Erosive	4	Fluocortolone 0.25% in cream—twice daily	NA
7	1980	M	69	U	Burning	No	Parapsoriasis, Vitiligo, Chronic gastritis	No	5	Erosive	4 to 0	Clobetasol propionate 0.05%	Remission in 3 months
8	1986	M	64	U	Aesthetic complaints	No	No	No	36	Atrophic	2 to 3	Fluocortolone 0.25% in cream	Aggravated in 10 months
9	2022	F	35	U	Aesthetic complaints	No	No	No	24	Atrophic	3 to 0	Methylprednisolone aceponate 0.1%, hydrating lip balm	Remission in 5 months
10	2022	F	51	R	Aesthetic complaints	Yes	Hypothyroidism	Euthyrox	10	Erosive	5 to 0	Methylprednisolone aceponate 0.1%, hydrating lip balm	Remission in 11 months
11	2023	M	76	R	Pain	Yes	IBD, Prostatic hypertrophy	Statins, Antihypertensives	3	Erosive	4 to 0	Methylprednisolone aceponate 0.1%, hydrating lip balm	Remission in 3 months

Legend: M—male, F—female, U—urban area, R—rural area, IBD—inflammatory bowel disease; NA—not available.

**Table 2:** Analysis of the literature review.

Year	Reference	Country	No. of Patients	Age/Sex	Mean Age (Years)	Unique Lip Involvement	Main Symptoms	Clinical Features	Skin Involvement	Systemic Pathologies	Biopsy	Diagnosis	Treatment	Outcome
2023	Fujita [19]	Japan	1	54/M	NA	Yes	Pain	Erosive	Yes	Psoriatic arthritis	Yes	OLP	Topical miconazole nitrate, topical corticosteroid, topical tacrolimus	Improvement after replacing secukinumab with risankizumab 150 mg
2022	Mozafari [20]	Iran	1	45/M	NA	No	No	Keratotic, plaque	No	No	Yes	LPL	Tacrolimus 0.1% + Clobetasol	Regression, recurrence
2022	Mittal [21]	India	1	50/M	NA	Yes (LL, UL)	No	Plaque	No	NA	Yes	OLP	NA	NA
2022	Hasan [22]	India	1	53/M	NA	Yes (LL)	Tenderness, burning	Erosive	No	No	Yes	LPL	Turbocort 0.1%	Remission
2022	Lehner [34]	France	23	NA	67 (SD ± 9.1)	No	Pain	NA	NA	NA	NA	NA	Topical corticosteroid	Improvement (12), No improvement (2)
2021	Yanagihara [23]	Japan	1	45/F	NA	Yes (LL)	Pain	Plaque	No	No	Yes	LPL	Topical corticosteroids	Remission
2020	Neema [35]	India	12	NA	NA	Yes (9)	NA	NA	NA	NA	Yes	LPL	NA	NA
2020	Misra [36]	India	2	50/F, 22/F	36	No	Pain, bleeding	Erosive	Yes	NA	Yes	LP	Tacrolimus 0.1%, Amlexanox 5%, Levamisole 150 mg	Remission
2020	Economopoulou [24]	Greece	1	66/M	NA	Yes (LL)	Pain, bleeding	Erosive	No	Oral cavity cancer, Skin LP 4 y ago	Yes	Nivolumab-related LPL	Betamethasone cream	Remission
2020	Garma [37]	Tunisia	2	34/M, 33/F	33.5	Yes (LL)	Pain, bleeding	Erosive	No	Diabetes (male)	Yes	LPL	Clobetasol (male), Betamethasone female)	Remission (male), Regression and recurrence (female)
2019	Hasan [2]	India	1	50/M	NA	Yes (LL)	Itching, burning	Erosive	No	No	Yes	OLP	Kenacort 0.1%	Regression, recurrence
2018	Mathur [25]	Nepal	1	44/M	NA	Yes (LL)	No	Plaque	No	No	Yes	OLP	Betamethasone dipropionate 0.5%	Remission
2018	Yu [26]	China	1	38/F	NA	Yes (LL)	Pain, bleeding	Erosive	No	No	Yes	OLP	Traditional Chinese medicine	Remission
2017	Choi [27]	Singapore	1	62/F	NA	No	No	Erosive	No	No	Yes	LPL	Tacrolimus, Hydrocortisone	Remission
2016	Nuzzolo [1]	Italy	13	NA	71.85 (SD ± 6.72)	NA	Burning, pain	Erosive, keratotic, annular	NA	NA	NA	LP	Cortisone, Nystatin	Remission, stable, regression
2016	Morita [28]	Japan	1	63/M	NA	Yes (LL)	Pain	Erosive	NA	Hypertension, adrenal gland tumor	Yes	OLP	Topical corticosteroids	No improvement, malignization after 6 years
2015	Samal [29]	India	1	52/M	NA	Yes (LL)	No	Plaque	No	No	Yes	LPL	NA	NA
2012	Sugashima [30]	Japan	1	32/F	32	Yes (LL)	No	Annular, plaque, atrophic	No	Zinc allergy	Yes	LPL	Tacrolimus 0.1%	Remission
2012	Holmukhe [31]	India	1	40/M	40	Yes (LL)	No	Annular plaque	No	No	Yes	LPL	Tacrolimus 0.03%	NA
2010	Gencoglan [38]	Turkey	4	NA	51	Yes (LL)	Pain	Erosive, plaque	No	No	Yes	OLP	Imiquimod 5% cream	Remission (3/4); Regression and recurrence (1/4)
2007	Petruzzi [39]	Italy	10	NA	62.7 (SD 11.0)	Yes (LL, UL)	NA	Erosive, keratotic	NA	NA	Yes	LPL	Clobetasol propionate 0.05%, Tocopherol oil, Miconazole	Remission
2007	van Tuyll van Serooskerken [32]	Netherlands	1	75/F	75	No (LL)	Burning, bleeding	Plaques, bullous	Yes	No	Yes	OLP	Tretinoin 0.025%, Triamcinolone 0.1%	Remission
2005	Shichinohe [40]	Japan	2	64, 68/M	66	NA	Pain, bleeding	Erosive	NA	NA	Yes	LPL	Tacrolimus 0.1%	Remission
2003	Yu [33]	USA	1	44/M	44	Yes (LL)	Pain, burning	Erosive	No	Hypertension	Yes	OLP	Clobetasol propionate 0.05%	Remission

LL—lower lip; UL—upper lip; NA—not applicable.

## Data Availability

The data presented in this study are available on reasonable request from the corresponding author.

## Supplementary Materials

The following supporting information can be downloaded at: https://www.mdpi.com/article/10.3390/medicina60060987/s1.
